# “I Felt Somewhat Cheated and Deceived When that Roadblock Came”: PrEP-Related Medical Mistrust, Distrust, and Misinformation Among Black Sexual Minority Men

**DOI:** 10.1007/s10461-025-04892-8

**Published:** 2025-10-10

**Authors:** Kay A. Simon, Samantha E. Lawrence, Alyssa N. Clark, Veronica Hanna-Walker, Greyson Arnold, Redd Driver, Jolaade Kalinowski, Ryan J. Watson, Lisa A. Eaton

**Affiliations:** 1 Department of Family Social Science, University of Minnesota, 1985 Buford Ave, St. Paul, MN 55108 USA; 2 School of Social Work, University of Connecticut, Hartford, CT USA; 3 Department of Psychology, College of Wooster, Wooster, OH United States; 4 Department of Human Development and Family Sciences, University of Connecticut, Storrs, CT USA; 5 HIV Center for Clinical and Behavioral Studies, New York State Psychiatric Institute and Columbia University, New York, NY USA

**Keywords:** Medical mistrust, Medical distrust, Misinformation, Black sexual minority men, PrEP

## Abstract

Substantial advances have been made in medical technologies to reduce HIV transmission in previous decades. However, disparities regarding HIV transmission continue to persist, with Black sexual minority men (BSMM) disproportionately impacted. These disparities are driven by several different economic and socio-cultural barriers, some of which include the ongoing mistrust or distrust of medicine and science among communities of color. Furthermore, the spread of misinformation has also substantially hampered the ability to effectively communicate the importance and efficacy of a major tool that can be used to reduce HIV transmission: pre-exposure prophylaxis (PrEP). Therefore, the goal of this study was to provide an understanding of BSMM’s experiences with and trust in medicine and practitioners, more broadly. The current study utilized interview data to qualitatively analyze the experiences of 32 BSMM in the United States. A thematic analysis was conducted, and we developed three different overarching themes to categorize different aspects of (a) *medical mistrust*, (b) *medical distrust*, and (c) *misinformation*. Results suggest that mistrust, distrust, and the spread of misinformation pertaining to medical institutions, medications (e.g., PrEP, antiretrovirals), and practitioners are present and appear to influence perceptions of HIV/AIDS and interest, access, or uptake and use of PrEP. Findings can inform ways to effectively rebuild relationships with medical providers and communicate information about PrEP uptake and HIV/AIDS by addressing medical mistrust and misinformation.

As a result of advances in HIV treatment and prevention, the necessary tools to effectively stop the spread of HIV in the United States (U.S.) are available. The advancements of bio-behavioral approaches, particularly the development of pre-exposure prophylaxis (PrEP), provide an avenue to dramatically reduce HIV transmission rates. These approaches are especially needed among Black sexual minority men (BSMM) given that they are at elevated risk for contracting HIV compared to their White or heterosexual counterparts [1]. Notably, the increasing rates of HIV incidence in the Southern U.S. are primarily among this marginalized group of individuals, indicating a need for additional research to comprehensively understand the experiences of BSMM [2]. Current research suggests that 49% of new HIV infections occur in the Southern U.S., and that BSMM comprise 28.5% of new infections [3, 4]. However, a number of barriers to PrEP initiation exist that put BSMM at greater risk of contracting HIV, such as stigma, medical mistrust, or medical distrust [5]. Medical mistrust is a general wariness or suspicion related to healthcare workers or the overarching healthcare system [6], while medical distrust occurs due to previous negative experiences in these settings [5]. Although individuals from multiple groups may have varying degrees of medical mistrust, certain groups may have particularly high levels of mistrust of the medical system, such as BSMM. The potential for BSMM to report greater levels of medical mistrust is often rooted in historical and current mistreatment of BSMM in healthcare settings [7–9]. Thus, the goal of this study was to understand perceptions of PrEP use and access as related to HIV/AIDS or PrEP medical mistrust or distrust among BSMM.

Data from public health surveillance studies collected between 2018 and 2022 provide a complicated snapshot of HIV incidence rates—a decrease in new HIV infections between 2018 and 2020 was documented, followed by an increase to previous incidence rates after public health measures were removed [3, 4]. However, shifts in HIV incidence rates are not uniform, with significant disparities at the intersection of race/ethnicity, sexual, and gender identity, particularly in the Southern U.S [7]. Changes in HIV screening and detection, culture and lifestyle factors, and/or stigmatization of HIV may play a role in disproportionate HIV diagnoses. For example, greater inclusion of HIV testing spaces at community-based events has allowed for a more accurate assessment of incidence rates. In other instances, cultural and lifestyle factors, such as a longstanding and warranted history of hesitancy with the healthcare system among BSMM or homogeneous social and sexual networks may also be associated with elevated risk of contracting HIV [1, 6, 7, 9].

Although individuals in the Southern U.S. account for over half of new HIV infections, PrEP use in this region is proportionately lower than any other geographic region in the U.S., representing only 21% of all PrEP users [10]. Scholarship that utilized nationwide commercial pharmaceutical information in 2021 also indicated that uptake and use of PrEP is also low among BSMM, as this group makes up approximately 14% of PrEP users [10, 11]. Given the high incidence of HIV and suboptimal uptake of PrEP among BSMM in the Southern U.S., understanding the lived experiences that inhibit (or promote) PrEP uptake can critically inform efforts to engage BSMM in evidence-based prevention efforts crucial to ending the U.S. HIV epidemic. Because medical mistrust is elevated within Black communities [12], it may play a significant role in BSMM’s access to and use of PrEP. Previous studies indicate that medical mistrust contributes to the racial inequities seen in HIV-related outcomes [13–16]. Among people living with HIV (PLWH), a lack of trust in health care providers is associated with lower HIV treatment adherence [16] and this association is especially true among Black men living with HIV [13]. HIV-related medical mistrust may also manifest as conspiracy beliefs, or mistrust around the origin and treatment of HIV [14, 15], which is associated with a higher likelihood of condomless sex [17] but also increased HIV testing [14]. However, the association between medical mistrust and PrEP outcomes (e.g., access, uptake, and retention), among BSMM, has received less attention.

In the limited literature examining medical mistrust among BSMM, mistrust negatively impacts this group’s engagement with healthcare services. Associations have also been found between medical mistrust and longer gaps between medical exams [18], which may reduce opportunities for BSMM to access PrEP. Qualitative studies that have explored medical mistrust and PrEP suggest that sexual minority men may not disclose interest in PrEP because of concerns and perceptions of discrimination from healthcare providers [19]. Greater HIV conspiracy beliefs among BSMM is associated with a lower awareness of PrEP [20] and lower intentions to initiate PrEP use [21]. Although these associations are informative, it remains unclear how medical mistrust impacts BSMM’s access to and use of PrEP. Research on how medical mistrust surrounding PrEP manifests and is integrated into the lived experiences of BSMM is warranted. Furthermore, research on medical mistrust and associated constructs (i.e., medical distrust and misinformation) can help to identify and target effective areas for intervention to address how best to scale up use of PrEP among BSMM communities.

Medical distrust differs from medical mistrust in that mistrust is a general attitude of lacking trust in the medical system, while medical distrust actively questions the motives and trustworthiness of providers, often within historical, social or political contexts [22]. Medical misinformation is considered false information that is produced and disseminated that may or may not have the express intention of misleading those who consume it [23]. In addition to generally false information, misinformation can also include different aspects of beliefs related to surveillance by government institutions or other powerful bodies (e.g., the pharmaceutical industry or a personified medical industrial complex) as well as related conspiracy theories (e.g., HIV is a government created disease designed for population control [2]). Importantly, espousing or internalizing beliefs indicative of misinformation is distinct from experiences, or a clinical diagnosis, of paranoia in which an individual believes they themselves are targeted by these government institutions or bodies, among other diagnostic criteria [2, 23]. Even though HIV has been a health crisis for decades, there is still a large amount of medical misinformation that could contribute to a lack of PrEP uptake in BSMM. Misinformation in the form of inaccurate or false representations of HIV prevention medications (e.g., Truvada, Descovy, Apretude, Yeztugo), especially when occurring in tandem with stigma or medical distrust, may play a large role in PrEP uptake or ongoing use among BSMM [24–26]. Thus, the aim of the present study was to investigate how PrEP-related medical mistrust, medical distrust, and misinformation impact perceptions of PrEP use and access in a sample of BSMM in the Southern U.S. through the use of a combined deductive and inductive thematic analysis.

## Method

### Participants and Procedure

Thirty-two BSMM (*M*_*age*_ = 31.94; *SD*_*age*_ = 7.67**)** were recruited from the greater Atlanta, GA metropolitan area through targeted social media advertisements (e.g., Facebook and Instagram) and through participant referral. To participate in the study, individuals needed to report being (1) Black/African American, (2) 18 years or older, (3) assigned male at birth, and (4) report recent sex with a male partner. Individuals who identified as transgender or gender diverse were eligible for this study if they met other criteria; however, no participants in this study identified with a non-cisgender identity. Participants also did not need to be HIV negative, or a current or past PrEP user to be eligible to participate. Those meeting the eligibility criteria were recruited to participate in a qualitative study involving semi-structured interviews to inform the development of an HIV testing and PrEP uptake intervention program. Findings presented here are a secondary analysis of participant interview responses to provide a deeper understanding of individuals’ perceptions of PrEP and the broader medical system. For each interview, participants were read an Institutional Review Board (IRB) approved consent form by research staff prior to providing written informed consent. Following consent, interviews were conducted and transcribed. Interviews focused on participants’ experiences with PrEP use, healthcare systems, stigma, and attitudes toward HIV. Interviews lasted for approximately 40 to 50 min and occurred between February and April of 2019. The [REDACTED] IRB provided ethical approval for all study procedures. See [REDACTED] for additional information on demographic characteristics. Across the sample, 16 participants reported never having used PrEP (50%), followed by 10 participants who reported current PrEP use (31.25%), and six participants reported having never used PrEP (18.75%).

### Thematic Analysis

Data were analyzed via thematic analysis using a two-step coding process resulting in a combined deductive, then inductive, analysis. Specifically, we began our thematic analysis with a deductive approach in which preliminary themes were based on prior theory and literature [27] ahead of coding participant narratives [28]. Our major interest, and initial study goal, was to investigate participants’ experiences with the healthcare system and medical mistrust related to PrEP and HIV and thus two of the initial themes relied upon previous literature, medical mistrust and medical distrust [6, 8, 18, 29]. For example, a crucial distinction between mistrust and distrust in the literature is that distrust requires an individual to have experienced a negative interaction whereas mistrust is driven by social norms or beliefs surrounding the healthcare system within one’s own community [22]. Thus, when developing initial themes, this distinction was already present prior to deductive coding when investigating nuances between themes indicative of mistrust or distrust. Throughout this process, coders noted that participants continued to report beliefs indicative of misinformation, rather than more commonly held beliefs pertaining to medical mistrust (e.g., doctors take advantage of patients; the medical system cares more about money than healthcare). Following this, re-coding and the refining of initial themes occurred to generate *misinformation* as a third, and distinct theme from *medical mistrust* and *medical distrust*.

Following the refining of themes and addition of *misinformation*, we ensured that participant narratives aligned with these preliminary themes (i.e., *medical mistrust*, *medical distrust*, and *misinformation*) via an inductive approach which further characterized our themes to best represent participant experiences [30]. While experiencing negativity is a clear distinction between mistrust and distrust [22], relying on participant narratives to determine how related concepts might be characteristic of each of the three themes allowed us to effectively triangulate data to developed themes [31]. For example, understanding how conspiracy beliefs may better characterize one of our themes over another was driven by participant descriptions of beliefs. Throughout the data analytic process, participants highlighted nuances of misinformation which were distinct from broader beliefs of medical mistrust, or individual instances that produced medical distrust. Thus, misinformation was a theme developed from an inductive, rather than deductive, approach distinct from broader beliefs of medical mistrust and medical distrust.

To provide further rigor to our analyses, researchers documented all themes, details, and narratives that were substantial contributors to thematic development from the coding process into a codebook (i.e., memoing [30]). The coding team consisted of the second, third, and fourth authors who met weekly to reconcile any disagreements and refine themes, with the first author moderating the reconciliation process [31]. Coders analyzed a set number of interviews each week, independently, before meeting to resolve disagreements or refine themes as part of an iterative process, until all interviews were analyzed. Researchers calculated interrater reliability for each theme, as is common in thematic analysis procedures to encourage replicability as part of codebook development approaches. The three themes of *Medical mistrust* (K-α = 0.92), *Medical distrust* (K-α = 0.96), and *Misinformation* (K-α = 1.00), all showed excellent reliability [32, 33].

The coding team reflected a number of different backgrounds and experiences and proactively discussed reflexivity [27] and how these identities could influence analyses [34]. Specifically, coders discussed how their identities did and did not overlap with particants’ as well as their own feelings about the healthcare system, pharmaceutical industry, and science more broadly. Further, coders and the first author discussed ways in which negative experiences within medical settings changed their perspective on what distinguishes mistrust from distrust, and what constitutes misinformation. Some of the coders identified with sexual minority identities but did not share other identities with the participants. The first author attended coding meetings and holds some overlapping identities with the participants including being a sexual minority person of color and was raised in the Southern U.S. but does not explicitly identify as Black. Further, another member of the research team and author does identify as a BSMM and audited this coding process to ensure that this qualitative analysis authentically represents these participants’ experiences.ip

## Results

Three themes were developed through the research team’s qualitative analysis of the data. These themes are presented in order of which they were developed: (1) *Medical mistrust*; (2) *Medical distrust*; and (3) *Misinformation* (see Table 1 for additional theme descriptions and exemplar quotes, all participant names are pseudonyms to maintain participant confidentiality).

**Table 1:** Theme descriptions

Theme	Definition	Example	Exemplar Quote	K-α
Medical mistrust	References to prejudices, attitudes, or perceptions of social norms that are suspicious or wary of the healthcare/pharmaceutical system or providers	Believing that a drug is not effective due to previous misconceptions of medications or is profit-driven	“*I’m kind of just like*,* is this a scam? Is this one of those drugs that they’re going to have recalled…I don’t want to put anything in my body that’s going to be toxic*” *“I feel like they’re only trying to get a check and they’re not really concerned about healing"*	0.92
Medical distrust	Experiences that are confirming of medical mistrust such as discrimination or invalidation of one’s identity or healthcare needs	Feeling as if a doctor is treating you poorly or like an experiment	“*[I was] feeling like a ‘lab rat*,*’ they don’t understand the issues of… gay versus straight sex… some doctors just aren’t educated*” *“I don’t know. It’s kind of like the energy when I ask her - or*,* well*,* if I was to ask her something. I don’t know. I get that feeling where it’s like she’s not that excited or happy about ‘gay*,*’ quote*,* unquote stuff. Like*,* OK. It’s her job as a healthcare professional…"*	0.96
Misinformation	Broader descriptions of healthcare-related systems (or the government) that are unsubstantiated	Believing that the government is choosing not to cure HIV to generate money or to control the population	“*I think they have a cure for it [HIV]… they don’t want to release the cure… they lose money by curing it*,* so they can gain money by preventing it*” *“I don’t trust the government*,* one*,* because of population control*,* so who’s to say PrEP isn’t being disguised as something to kill off a whole bunch of people…*”	1.00

### Medical Mistrust

The first theme developed was *medical mistrust* which was characterized by statements that implied skepticism or hesitancy surrounding PrEP use and, thus, served as a barrier to PrEP use. Specifically, *medical mistrust* can be understood as a statement that implies a barrier to PrEP use that may have emerged due to general beliefs related to the healthcare system or pharmaceutical industry. Several different mechanisms contributed to the degree of mistrust that participants had such as one’s general attitude toward science, healthcare, or pharmaceuticals (e.g., hesitancy of results from clinical trials). For instance, Marcus (current PrEP user, HIV negative, 39 years old) noted that PrEP has not existed as long as other medications and thus the long-term effects might be unclear. Specifically, Marcus stated that:

But I don’t know that it’s been taken long enough, if there’s enough history and documentation on it and observation of PrEP patients to know really everything. You know? It could turn out 10 years from now, we find out that yes, PrEP stops you from getting HIV, but it gives you liver cancer.

Another concern that participants reported was that they might be treated as a ‘number’ or that providers are only treating patients for money. For example, Isaiah (past PrEP user, HIV negative, 29 years old) stated that “*It makes me feel like I’m just another check*,* you know? I’m just another dollar sign… I’m not important*.” Finally, some participants described narratives that may have been informed by the historical mistreatment of Black people. For example, Kasim (current PrEP user, HIV negative, 34 years old) talked about how Black men often do not go to doctors and that he, as a Black gay man, has started to break through this stereotype. Specifically, he stated that:

I think that a lot of guys don’t go to the doctor, especially men of color. We don’t go to doctors. We don’t go very often. Growing up, I didn’t see my father go to the doctor like at all. And that [is] something [that] is really, really wrong. So, I think that carries across the board, especially with men of color. Black men in particular don’t have great relationships with physicians quite often, so I don’t see – well I take that back. I was going to say I don’t see that being much different with gay men, but I do think that gay men sort of breakthrough that stereotype somewhat.

Our qualitative analyses suggest that narratives of *medical mistrust* emerge through several different socio-cultural and historical contexts, such as the longstanding history of the medical system experimenting on Black individuals [9]. Other psychological mechanisms such as a resistance to changes in attitudes, the difficulty of breaking rigid stereotypes (e.g., Black men avoiding doctors), or HIV-related information avoidance [35] also appear to emerge in the context of medical mistrust. This is indicative of the depth of mistrust that currently exists among marginalized individuals as it relates to the healthcare system and science. Overall, twelve of thirty-two participants described experiences that were coded as part of the theme *medical mistrust*.

### Medical Distrust

The second theme developed, *medical distrust*, was characterized by negative experiences with healthcare providers or settings that led to the development of distrust or concerns about future mistreatment. *Medical distrust* was developed as a theme distinct from *medical mistrust* because direct negative experiences shaped participant narratives differently from sources of mistrust. For example, Isaiah (past PrEP user, HIV negative, 29 years old) described not having enough funds for PrEP and how his insurance no longer paid for his prescription due to moving states and changing coverage. When he was unable to get a prescription refill, he specifically described how none of the pharmacists at the time were interested in providing any kind of resource or information with which he could solve this issue. Specifically, Isaiah in reference to the pharmacy/pharmacist said:

I really just felt useless, like I had no say-so, I’m glad it was just like some PrEP, and it wasn’t like an antibiotic or a prescription that would keep me alive because it’s like, damn, you would have let me die. I don’t have $5,000 or whatever thousand dollars to give you for this prescription to help me.

Importantly, this experience created one of several barriers to PrEP and the participant ultimately discontinued PrEP because of these healthcare- and insurance-specific barriers.

Holding the belief that an individual who is representative of the medical system (i.e., pharmacist) would rescind care dramatically shaped Isaiah’s experiences. Other participants noted how initial mistrust of a healthcare provider was later confirmed and turned into distrust. For example, Derreck (never used PrEP, HIV negative, 39 years old) described a situation where he was concerned that his doctor was not supportive of his sexuality, leading him to be uncomfortable asking for more information about PrEP (see Table 1). When Derreck did ask for information on PrEP, he felt as if he was an afterthought and stopped seeking care. Specifically, Derreck noted that “*It just felt like she didn’t want to be bothered with it*,* I guess*,” and would later change providers in part due to this experience. Participants also described experiences of stigmatization that would contribute to their distrust of the healthcare system. For instance, when Andre (current PrEP user, HIV negative, 25 years old) was describing barriers to PrEP access, he pointedly acknowledged the role that doctors play in stigmatizing sex while also requesting participants be transparent about their health:

I’m like, you just asked what type of sex I have or what am I doing, and then you frown and look crazy. It’s like, why are you doing the work that you do? Why are you working in a PrEP HIV/STD clinic if you’re not comfortable talking about other people’s sex? That’s a big portion of what you do in your job. So, I think that was a part of my experience too.

The impact of these negative experiences such as feeling useless, invalidated, stigmatized, disempowered, or ignored by society shaped participant experiences and led to the perception that barriers to PrEP access were all but insurmountable for many participants. This, in turn, furthered participants’ beliefs that the healthcare system would inevitably fail or harm them which developed into a defining characteristic of *medical distrust*. For some individuals, healthcare professionals are perceived as a barrier to HIV-related care and access to PrEP, which is reflected in participant responses, with Andre’s experience being of particular concern. That Andre experienced judgment from a medical provider employed at a clinic specializing in HIV- or PrEP-related care highlights that stigma is pervasive across many contexts, even in spaces designed to be culturally responsive. Eleven of thirty-two participants described experiences that were coded as being part of the *medical distrust* theme.

### Misinformation

Our third and final developed theme, *misinformation*, was characterized by believing factually inaccurate PrEP-, HIV-, or healthcare-related information that an individual may report with no reputable source to support this claim. In particular, *misinformation* as a theme was characterized by statements that substantially deviated from reputable sources or were directly relevant to a participant’s perceptions of PrEP. That is, *misinformation* varies from incorrect interpretations of information (e.g., interactive toxicity beliefs) to conspiracy beliefs (e.g., the government developed HIV) which created a barrier to PrEP initiation. For example, some participants noted that inconsistent PrEP use for individuals who were currently HIV-negative would lead to greater risk of contracting HIV or intolerance to the medication they were taking.

Notably, we did not apply a *misinformation* code to responses that may now be considered incorrect, but (a) could reasonably be true or may have been accurate at the time of the data collection (i.e., February through April of 2019) such as the belief that PrEP was only 93% effective at preventing HIV transmission or (b) a widely believed narrative within participants’ own community, such as the belief that PrEP will always damage one’s liver or that PrEP and PEP (i.e., post-exposure prophylaxis) were the same prevention strategy. We chose not to include these descriptions as *misinformation* to differentiate between instances of misunderstanding or being misinformed due to a lack of information and broader understandings of misinformation (i.e., inaccurate information that contributes to one’s worldview). However, severe instances of *misinformation* did emerge among participants such as conspiracies, extensive feelings of suspicion, or other perceived threats that may have resulted from or reinforced *misinformation*. For example, Kevin (current PrEP user, HIV negative, 25 years old) noted that HIV and cancer were created by the government and that PrEP is a stop-gap measure. Although still a PrEP user himself, Kevin stated:

I said PrEP is for now [the best option], but it’s not the answer. And the reason why I say that is because honestly, I believe that HIV and cancer is man-made, and the reason why I say that it’s man-made is because a lot of things is through the government.

Similar widespread government conspiracies were shared among participants, some of whom mentioned that PrEP may be used as a form of population control (see Table 1). Other misinformed beliefs also characterized the theme *misinformation* such as how PrEP, along with other medications, function to reduce HIV transmission or maintain the health of others. For example, Jay stated that PrEP is “… *a placebo and that as long as you are able to convince someone that this is what this is supposed to do*,* then I think that the brain will allow your body to perform it*,* so it’s almost like a sugar pill*”. Thus, although Jay’s beliefs were not driven by far-reaching skepticism or structural harm, such as Kevin’s beliefs, this misinformation still discouraged PrEP uptake. Overall, six of thirty-two participants described experiences that were coded as *misinformation*.

## Discussion

The narratives presented in the current study among BSMM residing in the Southern U.S. demonstrate how medical mistrust, distrust, and misinformation qualitatively manifest and impact PrEP engagement to prevent HIV. Although there is little work that investigates the role of medical trust as it relates to HIV or PrEP [29], our findings suggest that medical mistrust, distrust, and misinformation serve as significant barriers to PrEP uptake and engagement with the medical system. Specifically, without explicit prompting, many participants in our study reported that trust in the healthcare system or in practitioners was a major barrier to PrEP uptake. As part of these explanations, we were able to comprehensively develop the themes *medical mistrust*, *medical distrust*, and *misinformation* through a mixed inductive and deductive thematic analysis. Broadly, these findings advance our understanding of how PrEP can be integrated into the broader literature on mistrust of health care institutions, providers, and medication, as well as the ways in which PrEP may be distinct from medical trust that occurs in other health contexts (e.g., all medications are bad compared to PrEP as a specific medication to mistrust or distrust).

Our first developed theme, *medical mistrust*, was characterized by reports of prejudicial attitudes, a deep investment in social norms, or the acknowledgement and/or internalization of cultural history involving the mistreatment of Black communities. Participants were also coded as being part of the *medical mistrust* theme when pushing back against deeply ingrained social norms. For instance, one of the participants reported that, as a general rule, Black men do not go to see the doctor for several different reasons (e.g., masculinity; cultural contexts) and that his choice to visibly interact with the healthcare system was an important way of being a role model. These different characteristics of *medical mistrust* are also reflected in some parts of the literature which finds that peers and role models are an important method of cultural transmission and can impact trust of the healthcare system for individuals and related social networks [12]. This cultural transmission, or socialization, is an important mechanism through which communities are held together, and intergenerational culture is fostered [36]. Furthermore, many of these messages among marginalized communities, including those related to medical mistrust are a product of deep harm that has been enacted on these groups. Restorative justice and rebuilding trust with marginalized communities will be an integral part of changing narratives of cultural socialization that can acknowledge past harms while also imparting values that encourage promotive behavioral health practices (e.g., not avoiding HIV testing centers; interacting with healthcare providers more often [35]).

Interestingly, we did not find many references to patient-provider racial concordance (or lack thereof) as a potential driver of *medical mistrust*. Rather providers were often referenced in the context of treating participants like a ‘number’ or ‘just a check’ with a perception of practitioners as only being invested in generating money and not medical care. While racism was not specific to patient-provider relationships, it did seem that racism was integral to defining the nuances in social norms. For example, there is a deep history of the scientific and medical community experimenting on Black individuals [9], so a legacy of hesitancy related to unknown long-term side effects of medications is expected [12, 14, 20]. One explanation for the absence of this finding is that broader medical mistrust is distinct from mistrust specific to PrEP or HIV. While structural racism is well-known to contribute to medical mistrust and a lack of engagement with the healthcare system among Black communities, PrEP and HIV-related stigma may play a distinct role. If interpersonal relationships pose a significant barrier to PrEP uptake or HIV testing, racial but not sexual identity concordance between BSMM and their healthcare providers may lead to greater fears of judgment or stigmatization [37]. Increasing the number of community-based healthcare educators or workers who are also BSMM could serve to reduce medical mistrust specific to PrEP or HIV, in addition to mistrust of the broader healthcare system.

Our second theme, *medical distrust*, was characterized by negative experiences with individuals affiliated with the healthcare system (e.g., doctors, nurses, pharmacy workers) such as not receiving help when seeking care, being judged, or asked questions perceived to be invasive, by providers; or experiencing overt discrimination. Furthermore, experiences related to the theme of *medical distrust* often served as a major barrier to seeking care that participants believed was insurmountable. For instance, one participant, in addition to others described previously, mentioned that their doctor would rescind care entirely and stop prescribing PrEP if the participant continued to be engaging in “too much sex,” even though PrEP itself is designed as a preventative or harm reduction tool for sexually active people. Thus, participants continued to develop perceptions that healthcare-related individuals functioned largely as gatekeepers to PrEP, rather than people who could improve their health. Unfortunately, explicit negativity that generates medical distrust leading to even greater avoidance of the healthcare system which could lead to disinterest in PrEP and even a reduction in HIV testing.

As with *medical mistrust*, racial concordance did not seem to be a primary concern for participants in the context of *medical distrust*. Rather, if distrust was reported, participants often attributed their distrust to homophobic experiences, intentional ignorance, or sexual stigmatization and not explicitly tied to racism. One possibility for the lack of reference to experiences of racism might be that many individuals described interacting with community- or HIV-serving clinics which may be particularly committed to training staff in cultural competency or sensitivity [19, 37]. Future research should continue to investigate the role of identity concordance (e.g., a Black gay patient and Black gay provider) in healthcare settings given that other studies have found racism and sexual stigmatization to be a common barrier to receiving appropriate care [37, 38]. Another possibility is that while some negative interactions are driven by racism, specific experiences of homophobia, ignorance, or sexual stigmatization are more salient given the specific context in which these interactions are occurring [39].

Our third and final theme was *misinformation*, which was characterized by beliefs about PrEP, HIV, or healthcare that were factually inaccurate and with no reputable source to support a participant’s claim. For instance, some of the participants in the *misinformation* theme reported interactive toxicity beliefs (i.e., the combination of medications leading to a negative health experience [40]) or misunderstandings of PrEP as a medication (e.g., that an individual is required to take PrEP for life). PrEP, HIV, and health-related literacy is relatively poor in the U.S., with many individuals reporting a lack of accurate health-related knowledge [41, 42] which could be a contributing factor to ongoing misinformation. Although these beliefs do serve as a barrier to interest or uptake of PrEP, they are potential targets for intervention.

Unfortunately, there were other participants who reported a more substantial degree of misinformation with beliefs that could be considered a conspiracy belief or were particularly inaccurate or harmful. Indeed, participants including Jay, noted that belief in a medication is a crucial aspect to successful treatment, rather than the effects of the medication itself (e.g., medication is only impactful if one believes in the efficacy of said treatment), such that even if PrEP were a sugar pill, his body would still protect him from contracting HIV. These severe instances of misinformation are likely especially difficult targets for intervention related to science education [20, 39], indicating a need for alternative interventions for people with particularly concerning misinformation beliefs [20]. One possible way to target this group of participants is to heavily emphasize the benefits of PrEP despite the inaccurate beliefs that an individual might have as an incentive for uptake. For example, one participant (Kevin), stated that the benefits of PrEP were especially important as it protected him from HIV, which he believed to be man-made and controlled by the government. For Kevin, the benefit of PrEP outweighed the negative associations with the healthcare system and was perceived as the ‘best option’ at his current life stage. Another possibility to reduce medical mistrust and encourage PrEP uptake is to improve public health communication and combat stigma at the individual, interpersonal, and structural level simultaneously, as has been suggested by prior research [43]. At the individual level, a lack of awareness and misunderstanding of one’s risk of contracting HIV are targets for intervention. Furthermore, generating public health messaging that focuses on reducing potential stigmatization of PrEP use within interpersonal relationships, such as from peers or partners, is another target for intervention. Finally, from a structural perspective, improving perceptions of local health departments will also increase trust between government bodies and BSMM communities, thereby reducing medical mistrust and improving PrEP uptake. While there are other barriers to PrEP uptake such as those related to finances, insurance, provider access, and transportation [1, 35, 37, 41, 43], efforts to improve science education and outreach are more amenable to timely interventions.

This work, as with any research study, comes with several limitations and strengths. Although this research did elicit some responses related to medical mistrust, distrust, or misinformation, the study was intended to inform an HIV testing intervention study and thus did not pointedly focus on an individual’s trust in the medical system more broadly (i.e., no questions specifically asked about medical mistrust, distrust, or misinformation). Interviews that comprehensively investigate beliefs of mistrust, experiences leading to distrust, and sources or perceptions of misinformation could provide additional narratives not represented here. However, that these three developed themes (i.e., *medical mistrust*, *medical distrust*, and *misinformation*) appeared to be distinct and substantial barriers to PrEP access or PrEP use without specific prompting does underscore the saliency of these constructs as barriers to HIV testing or PrEP uptake. Finally, recruitment largely occurred in Atlanta, GA, which could limit the generalizability of these findings to other states in the Southern U.S., or the U.S. as a whole.

## Conclusion

This work contributes to the ongoing literature [6, 12, 14, 15, 18, 35] on BSMM’s relationship to the healthcare system and medical mistrust, particularly in the context of perceptions and access of PrEP and/or HIV. Furthermore, our findings highlight the importance of medical mistrust, medical distrust, and misinformation, as distinct barriers to PrEP access and/or uptake, and as barriers that are relatively common. Finally, our developed themes can also inform different types of interventions and barriers to PrEP at the individual, interpersonal, and structural level. Future research should investigate ways to effectively reduce beliefs of mistrust, experiences of distrust, or persistent misinformation that appears in communities related to HIV, PrEP, and the healthcare system.
